# Effects of Xylitol and Sucrose Mint Products on *Streptococcus mutans* Colonization in a Dental Simulator Model

**DOI:** 10.1007/s00284-017-1299-6

**Published:** 2017-07-17

**Authors:** Krista M. Salli, Ulvi K. Gürsoy, Eva M. Söderling, Arthur C. Ouwehand

**Affiliations:** 1 DuPont Nutrition and Health, Global Health and Nutrition Science, Danisco Sweeteners Oy, 02460 Kantvik, Finland; 2 Department of Periodontology, Institute of Dentistry, University of Turku, Lemminkäisenkatu 2, 20520 Turku, Finland

## Abstract

Few laboratory methods exist for evaluating the cariogenicity of food ingredients. In this study, a dental simulator was used to determine the effects of commercial sucrose and xylitol mint products on the adherence and planktonic growth of *Streptococcus mutans*. Solutions (3% w/v) of sucrose, xylitol, sucrose mints, xylitol mints, xylitol with 0.02% peppermint oil (PO), and 0.02% PO alone were used to test the levels of planktonic and adhered *S. mutans*. A dental simulator with continuous artificial saliva flow, constant temperature, and mixing was used as a test environment and hydroxyapatite (HA) discs were implemented into the model to simulate the tooth surface. Bacterial content was quantified by qPCR. Compared with the artificial saliva alone, sucrose and sucrose mints increased the numbers of HA-attached *S. mutans*, whereas xylitol decreased them. Similarly, planktonic *S. mutans* quantities rose with sucrose and declined with xylitol and xylitol mints. Versus sucrose mints, xylitol mints significantly reduced the counts of HA-bound and planktonic *S. mutans*. Similar results were observed with the main ingredients of both types of mints separately. PO-supplemented artificial saliva did not influence the numbers of *S. mutans* that attached to HA or planktonic *S. mutans* compared with artificial saliva control. In our dental simulator model, xylitol reduced the counts of adhering and planktonic *S.mutans*. The mints behaved similarly as their pure, main ingredients—sucrose or xylitol, respectively. PO, which has been suggested to have antimicrobial properties, did not influence *S. mutans* colonization.

## Introduction

In the oral ecosystem, there is a complex, continuous interaction between dietary constituents, the oral bacteria, teeth, and saliva. A food or a constituent of it can contribute to the formation or development of caries through two common routes: inducing defects on the texture of the tooth, for example, by decreasing the pH, and altering the composition of healthy oral biofilm, which increases the number and virulence of caries-associated bacteria [17]. Sugars and syrups (consisting of mono-, di-, and oligosaccharides) are added to foods, usually as sweeteners, preservatives, or functional components. One such sugar, sucrose, is highly associated with the risk of caries with regard to its amount in food and the frequency of its consumption [17]. A recent review confirmed these findings and suggested that lowering sucrose intake to below 10%E (percentage of daily energy) mitigates the risk of caries and that any further reduction to below 5%E has additional benefits [27].

Because the consumer still enjoys sweet-tasting food, alternative sweeteners, such as polyols, have been recommended to replace sucrose in food products. Xylitol is a five-carbon polyol, that is isosweet to sucrose. It is non-cariogenic and has beneficial effects on oral health [11, 28]. Xylitol is not fermented by mutans streptococci (MS) and reduces their numbers and growth; it also decreases the amount of plaque [25, 38]. MS consists mainly of *S. mutans* and *Streptococcus sobrinus* [22]. Xylitol is used in confectionary and dental care products. Many consumer products with xylitol also contain mint extracts.

Essential oils are another group of commonly used food additives. These compounds are often considered natural antimicrobials and have been proven to suppress planktonic growth and biofilm formation by some oral microorganisms [16, 34]. However, essential oils are not a homogenous group and various oils need to be separately evaluated with regard to different applications, like oral biofilms. Although peppermint oil is used widely in chewing gums, oral rinses, and pastilles, there is little information on the effects of mint products on oral bacterial biofilms [13]. Only few studies have demonstrated that the formation of 17h batch biofilm on polystyrene tubes by *S. mutans* was inhibited by peppermint oil, at 6000 ppm [31, 35].

Sugars, such as sucrose, starch, fruits, and dairy products have been analysed with regard to their cariogenic properties [8, 23, 26]. Several models, with mono-species, multi-species, or microcosm bacteria, have been used to examine the caries-related effects of sucrose [1, 6, 10, 21, 37, 40] and xylitol [2, 14, 24]. When comparing the cariogenicity between sucrose and other products, in vitro methods are valuable, providing ethical and reproducible means to evaluate the attributes of these products. However, such methods merely evaluate the factors that affect caries development, not the multifactorial disease itself. Recently, a dental simulator model that delivers a continuous flow of artificial saliva was introduced to analyse the cariogenicity of various food components [3, 12, 32]. This model mimics salivary flow, in contrast to many batch culture models [2, 14, 21, 24, 32], and includes a solid surface that is composed of hydroxyapatite to simulate teeth [12, 32, 33]; making it superior to available chemostat models [5].

The aim of this study was to examine how *S. mutans* colonization is affected by sucrose- and xylitol-containing mint products in an in vitro dental simulator. We also evaluated the main ingredients of the mint products, sucrose, xylitol, and peppermint oil (PO), to determine whether there were synergistic effects between xylitol and mint. The applied simulator model was described earlier; however, commercial mint flavoured pastilles and appropriate sugar-based control pastilles were tested for the first time in this present study. Adhering and planktonic bacteria were quantified using molecular techniques.

## Materials and Methods

### Microorganisms and Growth Conditions

*S. mutans* ATCC 25175 (DSM 20523) was used as the model organism. Bacteria were cultured per Salli et al. [32]. Before the simulation, a fresh culture was prepared in brain–heart infusion broth (LAB049, LabM Limited, Lancashire, United Kingdom) and grown to the midexponential phase (OD_600_ = 0.4–0.6, corresponding to approximately 6 × 10^7^ colony-forming units (CFU)/mL). The culture was centrifuged, washed once with artificial saliva, and diluted to one-fourth of the original concentration. Each simulation vessel was inoculated with 0.5 ml of the diluted culture.

### Test Compounds

Stock suspensions [20% (w/v)] of sucrose (Suomen Sokeri Oy, Kantvik, Finland), xylitol (DuPont, Kotka, Finland), commercial mint product with xylitol (Fresh Mints Peppersmith, Peppersmith Ltd, London, United Kingdom), and commercial mints with sucrose (POLO mints, Nestle UK Ltd, York, United Kingdom) were prepared under aerobic conditions in sterile water and sterilized by filtration (0.2-µm Minisart^®^, Sartorius AG, Göttingen, Germany). All ingredients dissolved easily in sterile water. The ingredients of the two commercial products are shown in Table 1. For the experiments, artificial saliva was prepared separately with 3% (w/v) sucrose, 3% (w/v) sucrose mints, 3% (w/v) xylitol, 3% (w/v) xylitol mints, 3% (w/v) xylitol with 0.02% (w/v) peppermint oil (PO), and 0.02% (w/v) PO, and plain artificial saliva was prepared as control. The solution of mint pastilles was 3% (w/v) with regard to their carbohydrate content (only xylitol in xylitol mints and sucrose, glucose syrup, and modified starch in sucrose mints) to allow xylitol and sucrose to be compared. Xylitol mints had 92 g xylitol of 100 g product and sucrose mints 98.1 g carbohydrates of 100 g product of which 95.6 g was sucrose.

**Table 1:** Ingredients of mint pastilles

Xylitol mints	Sucrose mints
Xylitol	Sugar
Gum arabic	Glucose syrup
Calcium stearate	Modified starch
Peppermint oil	Stearic acid
Carnauba wax	Mint oils

Xylitol mint pastilles contained 0.6% PO by weight. When the pastilles were dissolved in artificial saliva to make a 3% (w/v) solution, the PO level in the final solution was 0.02% (w/v). We used three 100% etheric peppermint oils (*Mentha arvensis L. var. piperascens Malinv. ex Holmes* and *Mentha arvensis L. glabrata (Benth.) Fern*. from SALUS Haus Gmbh & Co KG, Bruchmühl, Germany; *Mentha Piperita* from Emendo Oy, Vaasa, Finland; and *Mentha piperitae ex arvensis* from Urtegaarden Aps, Allingåbro, Denmark*)* in the experiments. PO was weighed and added to plain artificial saliva or artificial saliva with 3% xylitol, such that the levels of PO were 0.02% (w/v) in the final solution, as in the xylitol mint solution. The results of the three POs were combined, because there were no differences between them.

### Stimulated Saliva for Pellicle Formation

Stimulated saliva was collected as described earlier. In short, paraffin-stimulated saliva was collected from volunteers, pooled, filtered, centrifuged, pasteurized, and stored at −20 °C before use [3, 15].

### Dental Simulator Model

A dental simulator model was used to study the effects of sucrose and xylitol mints on *S. mutans* quantities [12, 32, 33]. The model comprises a chamber system of 16 bottles and uses artificial saliva as growth media [3]. Hydroxyapatite (HA) discs (Clarkson Chromatography Products, South Williamsport, USA) were used to mimic dental enamel of teeth. Artificial saliva was prepared per Björklund et al. [3]. This system is detailed in Salli et al. [32]. Prior to being inserted into the simulation vessel, the HA discs were coated with stimulated human saliva and kept at 37 °C for 1 h to form a pellicle. A bacterial suspension was used to inoculate the culture vessels (15 mL of artificial saliva) before the simulation. At the outset of the simulation, 10 mL/h artificial saliva alone was pumped through the system for 30 min. Test compounds in artificial saliva were then added for 3 h at 20 mL/h, followed by 30 min of incubation, and a final rinse with 10 mL/h artificial saliva for 1 h. The HA discs were collected, and samples of artificial saliva from the growth vessels were taken after the rinse. The samples were stored at −20 °C until use. Unsupplemented artificial saliva was used as a control.

### Quantification of Bacteria Levels

DNA was extracted from the HA discs per Wilson [41], modified as previously described [32]. DNA was resuspended in elution buffer (Ambion Inc., Austin, USA) and stored at −20 °C.

DNA from artificial saliva samples was extracted using MagMAX™Total Nucleic Acid Isolation Kits (Ambion Inc.) as per the manufacturer’s instructions with the Mag MAX ™ Express 96-sample preparation system (Life Technologies, Halle, Belgium). Bead beating was performed using a Precellys24 (Bertin Technology, Montigny le Bretonneux, France), and DNA concentrations were measured on a Nanodrop ND-1000 full spectrum UV/VIS spectrophotometer (Thermo Scientific, Wilmington, USA).

Bacteria were quantified by quantitative polymerase chain reactions (qPCR) on an Applied Biosystems Real-Time PCR system (ABI 7500 FAST, Applied Biosystems, Foster City, USA). The reaction contained Power SYBR Green Master Mix without AmpErase UNG (Applied Biosystems, Bridgewater, USA) and 300 nmol of each primer. The reaction volume was 25 µL and contained 1 ng of template DNA. The primers were Str 1 5′-GTACAGTTGCTTCAGGACGTATC-3′ and Str 2 5′-ACGTTCGATTTCATCACGTTG-3′ [30]. The amplification programme was as follows: 95 °C for 10 min and 40 cycles of denaturation at 95 °C for 15 s, annealing at 60 °C for 30 s, and extension at 72 °C for 30 s. To generate standard curve, a tenfold dilution series from 1 pg to 1 ng of the target species *S. mutans* strain ATCC 25175, was included in the PCR assay. Bacterial quantities were measured in triplicate samples, and the results were expressed as log10 genomes per mL artificial saliva or per HA disc, normalized to the size and the 16S rDNA copy number of the standard species genome.

### Statistical Analysis

All data were the result of two or more independent experiments. In every experiment, each treatment was examined in at least duplicates. Statistical differences between treatment groups were analysed by one-way ANOVA and Tukey’s multiple comparison test. The statistical analysis was performed using GraphPadPrism version 6.04 for Windows (GraphPad Software, La Jolla, USA). Comparisons between sucrose-containing products and xylitol products in the ratio of HA-attached to planktonic bacteria were made using two-sided non-paired Student’s *t* test (Excel in Microsoft Office 365 ProPlus). *P* values of ≤0.05 were considered to be significant.

## Results

**Fig. 1:**
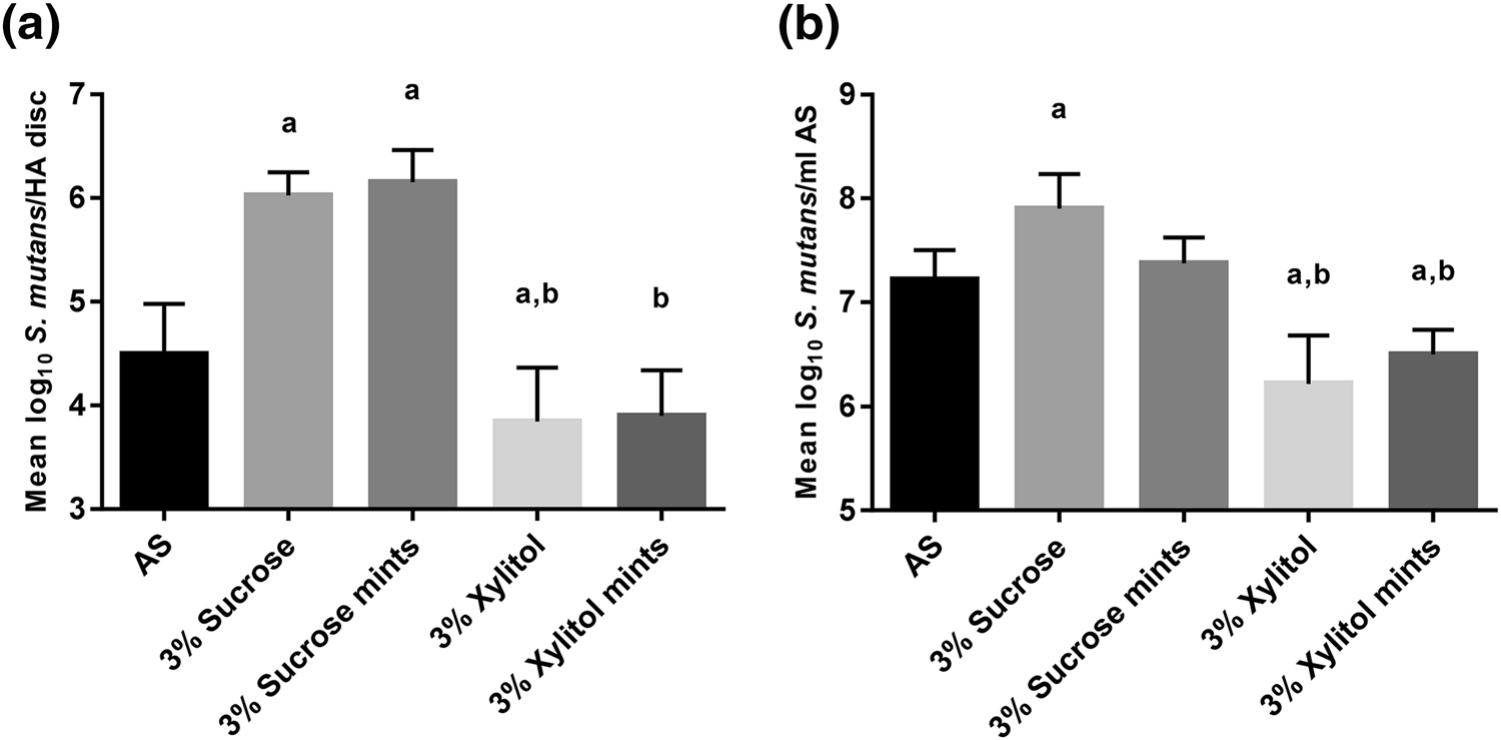
The effects of sucrose, sucrose mints, xylitol, and xylitol mints in artificial saliva (AS) on the numbers (mean and SD) of hydroxyapatite (HA)-attached and planktonic *S. mutans* in a dental simulator. DNA was extracted from **a** HA discs and **b** planktonic AS, and bacteria were quantified by real-time qPCR. Statistical significance (*P* < 0.05) is indicated by (*a*) compared with AS control and (*b*) compared with 3% sucrose and 3% sucrose mints

The results for sucrose, sucrose mints, xylitol, and xylitol mints are shown in Fig. 1. Compared with the artificial saliva control, the addition of 3% sucrose and 3% sucrose mints significantly increased the numbers of *S. mutans* that were attached to the HA (*P* < 0.0001, Fig. 1a). In contrast, 3% xylitol significantly decreased these counts (*P* = 0.019, Fig. 1a), whereas 3% xylitol mints had no significant effect (*P* = 0.14) versus the artificial saliva control. Moreover, the presence of xylitol and xylitol mints in the artificial saliva resulted in significantly less attachment of *S. mutans* to the HA discs compared with sucrose and sucrose mints (*P* < 0.0001 Fig. 1a). There were no differences between 3% sucrose and 3% sucrose mints (*P* = 0.99) or 3% xylitol and 3% xylitol mints (*P* = 0.99) (Fig. 1a).

The results on the planktonic samples were similar to those of HA disc-attached bacteria. Sucrose (3%) significantly elevated the numbers of *S. mutans* compared with the artificial saliva control (*P* = 0.018, Fig. 1b), whereas 3% xylitol and 3% xylitol mints significantly decreased them (*P* < 0.0001 and *P* = 0.006, respectively, Fig. 1b). However, the difference between 3% sucrose mint and artificial saliva control did not reach significance (*P* = 0.94). Versus 3% sucrose, both xylitol products significantly lowered bacterial levels (*P* < 0.0001, Fig. 1b). Compared with 3% sucrose mints, 3% xylitol mints and 3% xylitol significantly decreased *S. mutans* content (*P* = 0.007 and *P* < 0.0001, respectively, Fig. 1b). There were no differences between 3% sucrose and 3% sucrose mints (*P* = 0.24) or 3% xylitol and 3% xylitol mints (*P* = 0.58) (Fig. 1b).

**Fig. 2:**
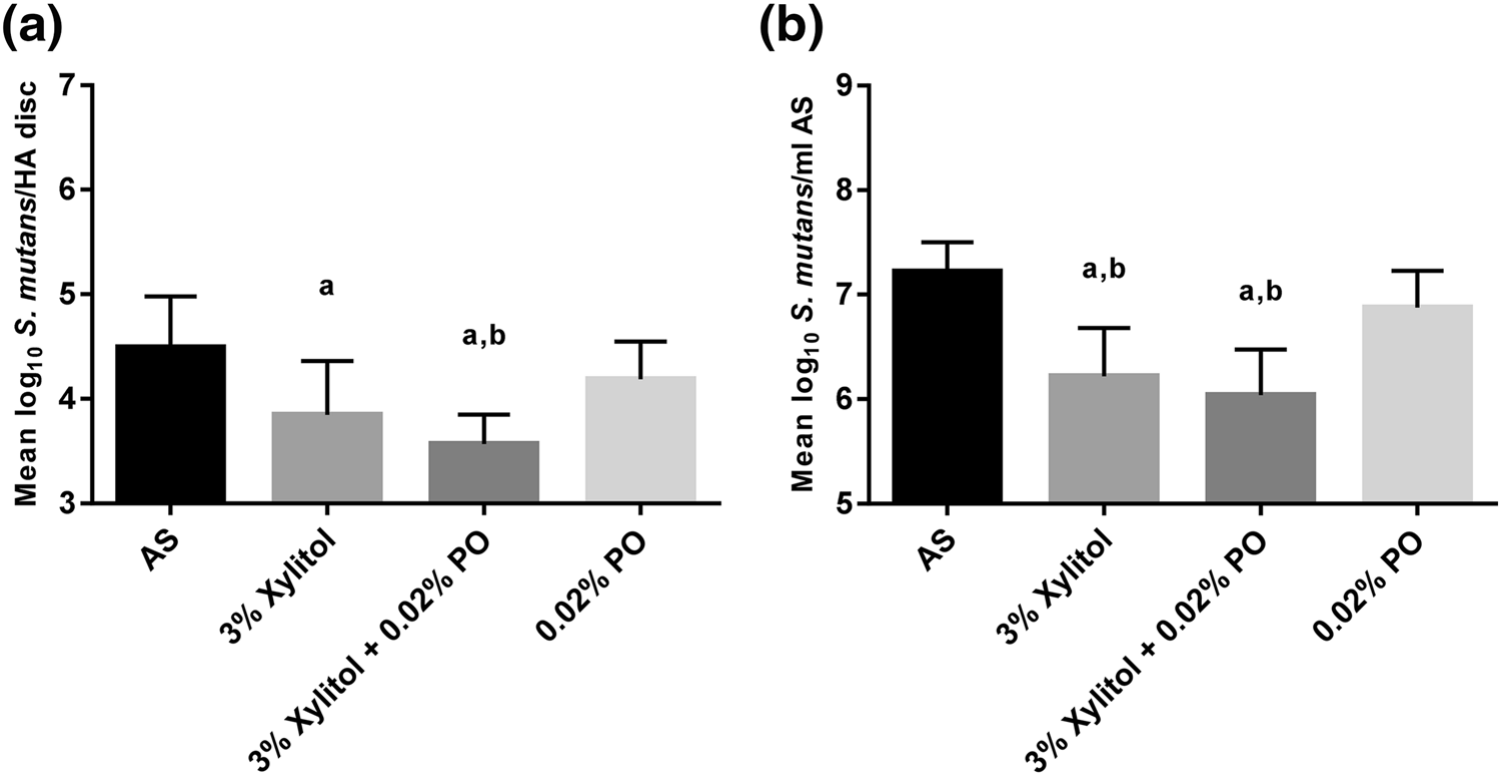
The effects of xylitol, xylitol with peppermint oil (PO), and PO in artificial saliva (AS) on the numbers (mean and SD) of hydroxyapatite (HA)-attached and planktonic *S. mutans* in a dental simulator. DNA was extracted from **a** HA discs and **b** from planktonic AS, and bacteria were quantified by real-time qPCR. Statistical significance (*P* < 0.05) is indicated by (*a*) compared with AS control and (*b*) compared with 0.02% PO

The results with xylitol, xylitol with PO, and PO are shown in Fig. 2. We tested three etheric POs, and because there were no differences between them, their results were pooled. PO alone did not have an effect on the number of HA-attached bacteria or planktonic *S. mutans* (*P* = 0.43 and *P* = 0.25, respectively, Fig. 2a, b) compared with artificial saliva control. The presence of 3% xylitol and 3% xylitol and PO in artificial saliva significantly lowered the numbers of *S. mutans* that adhered to the HA (*P* = 0.006 and *P* = 0.0001, respectively, Fig. 2a) compared with artificial saliva control. Versus PO alone, 3% xylitol with PO significantly decreased bacterial count on HA discs (*P* = 0.022), but 3% xylitol did not (*P* = 0.33, Fig. 2a). There was no difference between 3% xylitol and 3% xylitol with PO (*P* = 0.47) (Fig. 2a).

Compared with the artificial saliva control, 3% xylitol and 3% xylitol with PO elicited significantly less planktonic *S. mutans* (*P* < 0.0001, Fig. 2b). Planktonic *S. mutans* levels were also lower with 3% xylitol and 3% xylitol with PO versus PO alone (*P* = 0.005 and *P* = 0.0004, respectively, Fig. 2b). There was no difference between 3% xylitol and 3% xylitol with PO (*P* = 0.73) (Fig. 2b).

The ratio of HA-attached bacteria to planktonic bacteria was counted for all treatments (Table 2). The ratio of HA-attached to planktonic *S. mutans* bacteria was significantly higher for sucrose and the sucrose mints (3% sucrose 0.76 ± 0.03; 3% sucrose mints 0.83 ± 0.03) compared with xylitol, xylitol mints, and xylitol with peppermint oil (3% xylitol 0.62 ± 0.08; 3% xylitol mints 0.60 ± 0.06; and 3% xylitol + 0.02% PO 0.59 ± 0.06) (*P* = 0.0058).

**Table 2:** Comparisons of HA-attached and planktonic *S. mutans* bacteria (mean ± std)

Test compound added to AS	Log10 *S. mutans*/HA disc	Log10 *S. mutans*/1 ml AS	Ratio of mean HA/mean AS
3% Sucrose	6.0 ± 0.2	7.9 ± 0.3	0.76
3% Sucrose mints	6.2 ± 0.3	7.4 ± 0.2	0.83
3% Xylitol	3.8 ± 0.5	6.2 ± 0.5	0.62
3% Xylitol mints	3.9 ± 0.4	6.5 ± 0.2	0.60
3% Xylitol + 0.02% PO	3.6 ± 0.3	6.0 ± 0.1	0.59

*HA* hydroxyapatite, *AS* artificial saliva

## Discussion

Laboratory methods provide a simplified system for examining various aspects of caries separately, such as acid production, enamel demineralization, pathogen proliferation, biofilm development, and bacterial dysbiosis. In this study, we determined the effects of two commercial mint products on counts of planktonic and adhering *S. mutans* using an in vitro simulator that mimics the environment in the oral cavity. We observed that xylitol mints, like xylitol alone, impeded *S. mutans* growth and attachment, whereas sucrose products promoted them. PO did not affect *S. mutans* colonization.

The strengths of this study include its successful exploitation of an in vitro dental simulator model that measures adhering and planktonic bacteria for commercial mint products. The model focuses on the early steps of adhesion and biofilm formation; thus, a short biofilm formation time was used [12, 32, 33]. Young biofilm can be seen as clinically more relevant model, approximating normal oral hygiene. Apart from chewing gum, pastilles are the most commonly used consumer products with xylitol. Mint pastilles were selected for this study, because they are readily dissolved in artificial saliva and because there is limited knowledge on the combined effects of xylitol and PO on oral bacteria. This report is the first study to examine mint oils with this type of model. One limitation of the study was its use of a single bacterial strain. Although other oral bacteria are associated with dental caries, MS are linked to the development of caries [29, 39, 42]. In addition, high sucrose consumption is associated with elevated MS counts [18]. Polysaccharide matrix formation is essential for biofilm formation, and the polymers that are produced by *S. mutans* have high affinity to solid surfaces [4, 20]. *S. mutans* glycosyltransferases produce extracellular polysaccharides from sucrose and starch and *S. mutans* copes well with an acidic environment and environmental stress [4, 19, 43], prompting us to use *S. mutans* as the model organism in this study. Culturing only evaluates the number of bacteria that can be released from the biofilm and separated from each other. With qPCR, on the other hand, it is possible to quantify the total number of bacteria regardless whether they are single bacteria or aggregates.

We have observed that 2% xylitol in artificial saliva significantly decreases the numbers of both HA-adhering as well as planktonic MS [32]. Similar reductions in bacterial quantities were found in single-species (*S. mutans*) and three-species (*S. mutans, Streptococcus sanguinis*, and *Actinomyces naeslundii)* young batch biofilm with 5% xylitol [24]. For planktonic mixed oral bacteria in a 10-day chemostat, xylitol, pulsed with glucose, slowed acid production and prevented increases in *S. mutans* [5]. Our results are consistent with proposed mechanism of xylitol; impeding the growth and adhesion of *S. mutans* [38].

The results with 3% sucrose solutions in artificial saliva concur with what has been reported for 1% sucrose, a significant increase in the numbers of HA-attached bacteria with all tested strains [32]. MS ferment sucrose and use it as a substrate to produce extracellular polysaccharides [29]. In this study, we also counted the ratio of HA-attached *S. mutans* to planktonic *S. mutans* to all sucrose and xylitol test products. We then compared sucrose-containing test products to comparable xylitol-containing products. The ratio for sucrose and sucrose mints was significantly higher compared with xylitol and xylitol mints, indicating relatively less shedding and greater adhesion of *S. mutans* in the presence of sucrose.

Sucrose forms an important part of daily food consumption, thus elimination of sucrose-stimulated biofilms in oral cavity with xylitol pastilles is highly dependent of individual’s eating habits. However, there is clinical data showing that xylitol consumed in adequate mounts (5–6 g/day) three times a day along with normal diet, reduces the numbers of MS [38].

PO is often considered to be antimicrobial, but the scientific evidence is highly limited. Antimicrobial effects of PO are commonly examined by agar disc/well diffusion assay and by measuring the zone of inhibition. Two such studies did not report the antimicrobial activity of PO and Mexican mint on *S. mutans* ATCC 25175 [7, 9]. Conversely, another study found that high concentrations (1000–8000 ppm) of PO had antimicrobial activity and that 17-h *S. mutans* batch biofilm that formed on the walls of polystyrene tubes was inhibited by PO [31]. In our study, PO did not affect the planktonic growth of *S. mutans*. *S. mutans* grew as well in PO as it did without it. Further, at the concentration that we tested, PO had no effect on young biofilm formation, i.e. the bacteria on the HA discs. Our model evaluates the initial adhesion of bacteria to HA discs. The concentration of PO that we used in this study was as in the products commercially available (0.02% = 200 ppm). So, the peppermint oil concentration can be expected to be like what can be found in the oral cavity when using these products, and less than what has been studied by other groups. Biofilm is a unique structure of bacteria in the extracellular matrix that are in close proximity to each other, communicating, helping, and competing [36]. This mode of living affects many properties of bacteria, and in general, the bacteria in biofilm are often more resistant to antimicrobials. Interestingly, in HA-attached bacteria, the levels of *S. mutans* with xylitol and PO were significantly lower than those with PO alone, while this was not observed for xylitol alone. This indicates that xylitol and PO may have synergistic properties.

In conclusion, two commercial mint products were examined using a dental simulator model with regard to their effects on *S. mutans* growth and attachment to HA discs. Xylitol mint products did not promote growth or adhesion of *S. mutans*, like sucrose mints did. Xylitol induced the reduction of the numbers of planktonic and HA-attached bacteria compared with artificial saliva or sucrose. No additional benefit of peppermint against *S. mutans* colonization and proliferation was observed compared with xylitol or artificial saliva control.
